# Protocol for a randomized controlled dismantling study of an internet-based intervention for depressive symptoms: exploring the contribution of behavioral activation and positive psychotherapy strategies

**DOI:** 10.1186/s12888-019-2099-2

**Published:** 2019-05-03

**Authors:** Adriana Mira, Amanda Díaz-García, Diana Castilla, Daniel Campos, Sonia Romero, Juana Bretón-López, Azucena García-Palacios, Rosa Baños, Cristina Botella

**Affiliations:** 1 Universidad de Zaragoza, Teruel, Spain; 2 Universitat Jaume I, Castellón, Spain; 3 Universidad de Valencia, Valencia, Spain; 4 CIBER Fisiopatología Obesidad y Nutrición (CIBERObn), Instituto Salud Carlos III, Madrid, Spain

**Keywords:** Depression, Behavioral activation, Components, Randomized controlled dismantling study, Internet-based intervention, positive psychotherapy

## Abstract

**Background:**

There are evidence-based interventions for depression that include different components. However, the efficacy of their therapeutic components is unknown. Another important issue related to depression interventions is that, up to now, their therapeutic components have only focused on reducing negative symptoms rather than on improving positive affect and well-being. Because the low levels of positive affect are more strongly linked to depression than to other emotional disorders, it is important to include this variable as an important treatment target. Positive psychotherapeutic strategies (PPs) could help in this issue. The results obtained so far are consistent and promising, showing that Internet-based interventions are effective in treating depression. However, most of them are also multi-component, and it is important to make progress in investigating what each component contributes to the intervention.

**Methods:**

The current study will be a three-armed, simple-blinded, randomized controlled clinical trial with a dismantling design. 192 participants will be randomly assigned to: a) an Internet-based Global Protocol condition, which includes traditional therapeutic components of evidence-based treatments for depression (Motivation for change, Psychoeducation, Cognitive Therapy, Behavioral Activation (BA), Relapse Prevention) and PPs component, offering strategies to enhance positive mood and promote psychological strengths; b) an Internet-based BA Protocol condition (without the PPs component), and c) an Internet-based PPs Protocol condition (without the BA component). Primary outcome measures will be the BDI-II and PANAS. Secondary outcomes will include other variables such as depression, anxiety and stress, quality of life, resilience, and wellbeing related measures. Treatment acceptance and usability will also be measured. Participants will be assessed at pre-, post-treatment, 3-, 6- and 12- month follow- ups. The data will be analyzed based on the Intention-to-treat principle. Per protocol analyses will also be performed.

**Discussion:**

To the best of our knowledge, this is the first randomized dismantling intervention study for depression with the aim of exploring the contribution of a PPs component and the BA component in an Internet-based intervention. The three protocols are online interventions, helping to reach many people who need psychological treatments and otherwise would not have access to them.

**Trial registration:**

Clinicalstrials.gov as NCT03159715. Registered 19 May 2017.

## Background

Major depression is projected to become the largest contributor to the disease burden in high income nations by 2030 [1, 2]. Hundreds of controlled and comparative studies have examined the efficacy of psychological treatments for depression [3, 4]. Thus, we have evidence-based interventions for depression, including cognitive behavior therapy (CBT) [5, 6] and behavioral activation (BA) [7, 8], as well as interpersonal [3, 9] or problem-solving therapy [10]. Of all of them, CBT has been extensively researched as an intervention for patients with depression. Studies have shown robust evidence of its efficacy [3, 11–13]. Furthermore, the effect sizes found for CBT have been large compared to waiting list, placebo, or no-treatment conditions [14]. It usually consists of applied cognitive techniques to change thought patterns and behavioral techniques to activate behaviors [13]. However, the efficacy of its therapeutic components is unknown [15]. The literature shows that BA is an essential component of CBT treatments for depression [16–18]. Meta-analyses show that the effects of BA treatment on depressive symptoms are maintained or even improve at 6- and 12-month follow-ups, compared to control conditions and other treatments [7]. Nevertheless, less is known about how depression therapies work and the mechanisms that are responsible for their effects [19, 20]. The US Institute of Medicine indicated that a key step in being able to offer evidence-based interventions in clinical settings is the identification of the core elements of psychological interventions [21]. It has been pointed out that component studies are the best design to examine these core elements [15].

Another important issue related to depression interventions is that, until recently, their therapeutic components focused solely on improving negative symptoms (depressive symptoms, anxiety, etc.), and not on the promotion of positive affect, well-being, and character strengths [22]. Positive psychotherapeutic strategies (PPs) work to help in this issue by promoting positive functioning [22]. The main PPs are Well-being Therapy [23–26], which improves well-being dimensions based on Ryff’s model of eudaimonic well-being [27]; Quality of Life therapy [28, 29], which is more focused on promoting hedonic well-being and life satisfaction in various significant life domains; and Positive Psychotherapy [30, 31], focused on alleviating suffering and systematically enhance happiness by building positive emotions, strengths, and meaning in patients’ lives; and Strengths based Counseling [32, 33]. These interventions have been tested in clinical populations, particularly in depressed patients [34, 35]. They are effective in the improvement of depression and well-being [36, 37]. It has been established in the literature that depressive symptoms are related to lack of meaning in life and low levels of positive emotions, and that depression is more associated with low levels of positive affect than other emotional disorders [38]. Most of the positive interventions promote positive affect, gratitude, resilience, and positive functioning [22], and different studies have pointed out the importance of taking these variables into account as important depression intervention targets [39, 40].

Because depression is a prevalent mental disorder, one of the principal challenges nowadays is to develop new ways to deliver psychological interventions in order to maximize their efficiency and dissemination [41, 42]. Several internationally well-known research groups have launched Internet-based programs in an attempt to address this issue. Results obtained so far show that these online programs are effective in treating depression [3, 43–47]. However, as in the case of face-to-face interventions, most of the Internet-based intervention programs for depression are also multi-component, and it is important to make progress in investigating what each component brings to the intervention.

As mentioned above, component studies are an important tool for examining how therapies work, and they provide an appropriate way to identify the active elements of interventions [48]. With this study design, multicomponent treatments are decomposed, and the complete intervention is compared to an intervention in which a component is eliminated (dismantling studies) or to an intervention with an addition component (additive studies; [49]). There are few studies with a dismantling design in interventions for depression [50]. A recent comprehensive systematic review and meta-analysis of dismantling studies of psychotherapies for adult depression included only 16 studies, none with an Internet-based intervention [15].

Our research group has developed an Internet-based cognitive-behavioral treatment protocol that also includes PPs for patients suffering from depressive symptoms. It has shown its efficacy in different RCTs [51–53]. The results show that this intervention is effective overall, but we do not know the specific contribution of each of its therapeutic components. Using a dismantling strategy could help to identify the contribution of BA and PPs (its main therapeutic components) to the therapeutic change. As a result, this information can contribute to improving intervention programs for depression.

To the best of our knowledge no study with a dismantling design exists with an Internet-based treatment protocol for depression, and even less so with the specific objective of discovering the contribution of BA and PPs.

The objectives of the current study are to (a) evaluate the efficacy at post-treatment and follow-ups (3, 6, and 12 months) of a complete Internet-based protocol for depressive symptoms (including motivation, cognitive restructuring, BA, PPs, Relapse prevention), a protocol for depressive symptoms without PPs, and a protocol for depressive symptoms without a BA component, all of them administered through the Internet to patients with mild to moderate depressive symptoms; (b) analyze the mediators of change in depressive symptoms; and (c) study the acceptability and usability of the intervention.

## Methods/design

### Study design

The current study will be a three-armed, simple-blinded, randomized controlled clinical trial with a dismantling design. Specifically, three groups will be compared: a) an Internet-based Global Protocol condition (IGc), b) an Internet-based BA Protocol condition (IBAc), and c) an Internet-based PPs Protocol condition (IPPc). At this moment, the study is ongoing, and we are in the participant recruitment phase. Participants will be randomly allocated to one of the three experimental conditions.

Randomization will be stratified by levels of depression severity (mild to moderate).

**Fig. 1:**
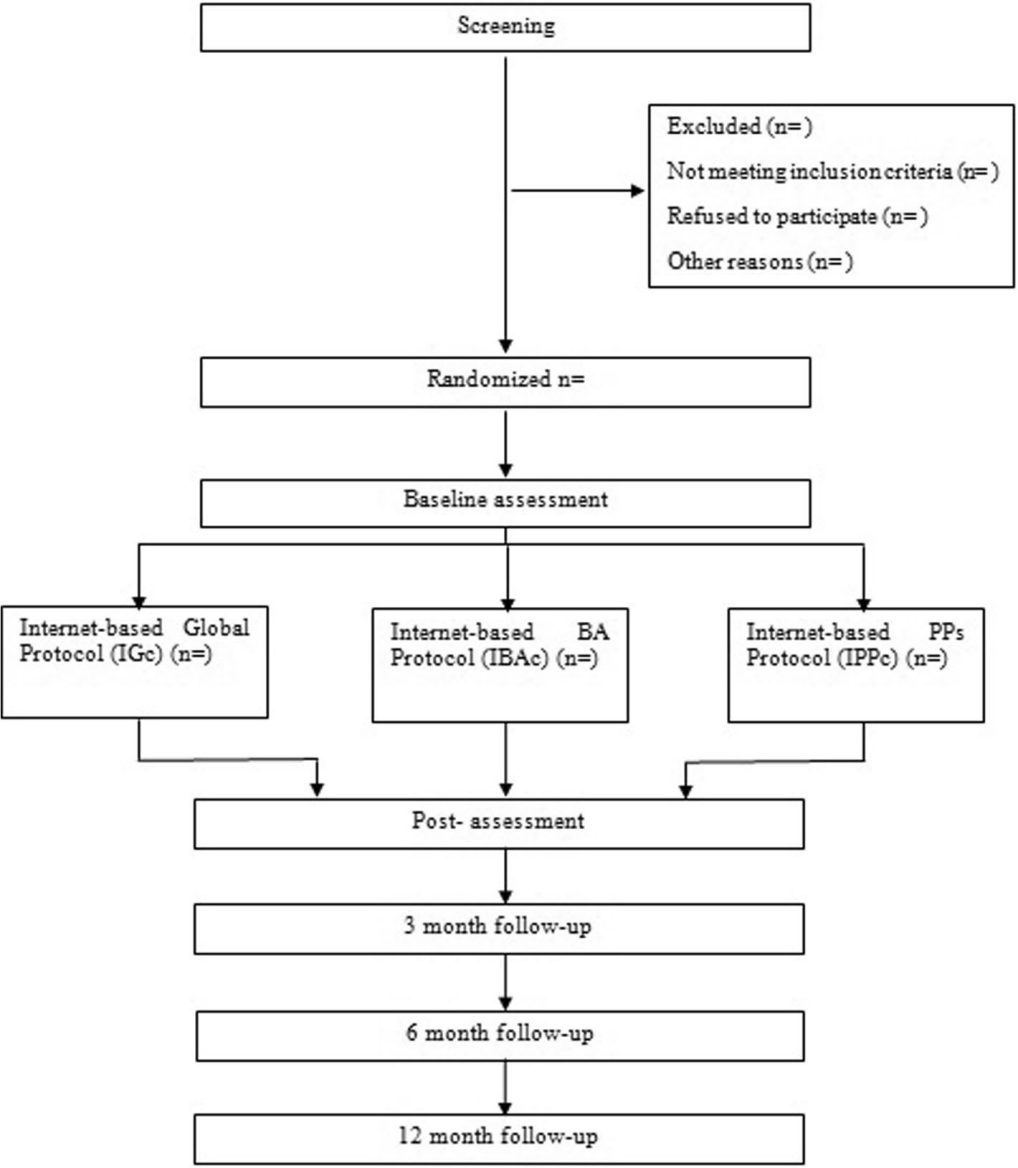
Study design

We will carry out block randomization within each stratum to certify that all levels of depression are balanced across the three experimental conditions. The study follows the CONSORT statement (Consolidated Standards of Reporting Trials) [54, 55], CONSORT-EHEALTH [56] and SPIRIT guidelines (Standard Protocol Items: Recommendations for Interventional Trials) [57, 58]. Participants’ assessments will be performed at pre-treatment and post-treatment. Furthermore, there will be three follow-ups: at 3, 6, and 12 months. Figure 1 shows the study flowchart.

### Participants, recruitment and eligibility criteria

The RCT will be carried out at the Emotional Disorder Clinic in Universitat Jaume I. Participants will be adult volunteers with mild to moderate depressive symptoms (from 14 to 28 on the Beck Depression Inventory-II, BDI-II) [59].

Participants will be recruited through the website specially developed for this aim, emails, phone calls, or personal visits, and they will be attended to by a clinician. Once a potential participant has been identified by the clinical psychologist, s/he will be informed about all the characteristics of the study. Any questions or doubts will be clarified in order to ensure that participants have understood all the information correctly. Those who are interested in participating will sign the informed consent. Furthermore, an independent researcher will assess whether they meet all the inclusion criteria.

To participate in the study, participants must meet these inclusion and exclusion criteria. The inclusion criteria are: a) 18 to 65 years old; b) able to read and understand Spanish; c) access to Internet; d) knowing how to use the Internet; and e) experiencing mild to moderate depressive symptoms (from 14 to 28 on the Beck Depression Inventory-II [BDI-II]). Exclusion criteria include: a) receiving a psychological intervention; b) suffering from a severe Axis I mental disorder: alcohol and/or substance dependence disorder, bipolar disorder, psychotic disorder, or dementia; c) having ideation or a significant plan for suicide (assessed by the MINI and item 9 of the BDI-II). Receiving pharmacological medication while participating in the study is not an exclusion criterion. However if the patient has an increase in the pharmacological treatment, s/he will be excluded from the study analysis. Nevertheless, a reduction in the medication does not imply exclusion from the study. We will offer alternative treatments to the participants who do not meet the criteria for the present study.

If the participants fulfill the eligibility criteria, they will be randomized to one of the three experimental conditions by an independent investigator who will not have information about the RCT’s characteristics. Randomization will be performed using a weighted random number sequence generated by a computer, in order to have a homogeneous distribution across the three conditions. The study researchers will be informed about the allocation schedule via telephone call. Before the allocation to one of the three interventions, the patients will agree to participate without having the information about which intervention they will be receive. Nevertheless, for pragmatic reasons, researchers and patients will not be blind to the intervention conditions. At any time during the study, the participants will be allowed to drop out of the intervention without giving any reasons.

### Ethics

This study will be carried out in conformity with the study protocol and the Declaration of Helsinki. Furthermore, it will guarantee the confidentiality and security of the data following the guidelines set forth in the current laws and data protection regulations: The General Data Protection Regulation, agreed upon by the European Parliament and Council in April 2016. Moreover, all important EU legislation and international documents on privacy will be followed.

All participants will be volunteers. When the study has been explained to them, they will sign the online informed consent to participate. Qualified clinical personnel will conduct the clinical assessment of the participants as part as the recruitment process. In the same way, all the tasks involving the participants will be performed by qualified, expert professionals (Clinical Psychology PhD). The assessment protocol is composed of standardized instruments (semi-structured interviews and questionnaires). Likewise, treatment protocols are based on empirically-validated treatments from the Task Force on the Promotion and Dissemination of Psychological Procedures of the American Psychological Association [60].

Data protection is an important aspect of the project. To access the Internet platform, the participants will have a unique username and password combination. Furthermore, the platform will be available on a 24/7 basis. Moreover, the transferred data will be secured using AES encryption (AES-256; Advanced Encryption Standard).

The study policies do not differ from those used in other clinical trials focused on the treatment of depression through the Internet, and so no special difficulties are expected.

The study has been approved by the Ethics Committee of University Jaume I (Castellon, Spain, approval number: 4/2017). The trial is registered at clinicalstrials.gov as NCT03159715.

Important protocol modifications will be communicated to relevant parties (i.e., trial participants, trial registries, journals, ethical committee and researchers).

### Interventions

#### Internet-based global protocol condition (IGc)

We have developed a manualized intervention protocol for depressive symptoms called Sonreír es Divertido (Smiling is Fun in English). It is an Internet-based program developed in a European project: Online predictive tools for intervention in mental illness [51]. Smiling is Fun includes the psychological treatment components that are traditionally incorporated in evidence-based interventions for depression: Motivation for change, Psychoeducation, Cognitive Therapy, BA, and Relapse Prevention. The program also incorporates a PPs component, offering tools to improve positive affect and promote strengths.

The intervention program includes eight modules: four CBT based modules; three PPs based modules, and one Relapse prevention module.

Regarding the CBT modules: 1) “*Motivation for change*”, 2) “*Understanding emotional problems*”, 3) “*Learning to move on*”, and 4) “*Learning to be flexible*”, their main objectives, respectively, are: a) To analyze both the advantages and disadvantages of changing their behaviors, feelings, and thoughts; b) To offer information to help the patient to understand the characteristics of the emotional problems; c) To explain the importance of being involved in life and acquiring an appropriate activity level; and d) To teach the patients how to think in a more flexible manner.

In the case of the PPs modules, designed to promote positive affect, well-being, and psychological strengths: 5) “*Learning to enjoy*”, 6) “*Learning to live*”, 7) “*Living and learning*”, their principal objectives are, respectively: a) To “savor” and enjoy positive life experiences; b) To do activities linked to their own goals and values and learn how to identify their psychological strengths; c) To know how to improve their psychological strengths and start working toward the future.

At the end of the program, there is a Relapse prevention component in module 8) “*From now on, what else…*?”, whose objectives are: a) To review what has been learned in each module; b) To learn that finishing the program does not mean no longer practicing the strategies learned, and that it is the beginning of each patient’s path; and d) To invite them to think about what they would like their future life to be like.

#### Internet-based BA protocol condition (IBAc)

This intervention protocol has these CBT components from the original protocol (*IGc*) explained above: Motivation for change, Psychoeducation, Cognitive Flexibility, BA, and Relapse Prevention. The PPs component is not included in this protocol.

The BA component in this protocol teaches the same things as the BA component in *IGc.*

#### Internet-based PPs protocol condition (IPPc)

This intervention protocol has these CBT components from the original protocol (*IGc*) explained above: Motivation for change, Psychoeducation, Cognitive Flexibility, and Relapse Prevention. The BA component is not included in this protocol.

The PPs component in this protocol teaches the same things as the PPs component in *IGc.*

Table 1 showed the structure of the IBAc and IPPc.

**Table 1:** The structure of the IBAc and IPPc

Internet-based BA Protocol condition (IBAc)	Internet-based PPs Protocol condition (IPPc)
Modules/ Therapeutic component	Modules/ Therapeutic component
1.“Motivation for change”/ Motivation	1.“Motivation for change”/ Motivation
2.“Understanding emotional problems”/ Psychoeducation	2.“Understanding emotional problems”/ Psychoeducation
3.“Learning to be flexible”/ Cognitive Flexibility	3.“Learning to be flexible” / Cognitive Flexibility
4.“Learning to be active”/ BA	4.“Getting involved with life”/ PPs
5.“My significant activities”/ BA	5.“Enjoying life”/ PPs
6.“Seeking support to be active”/ BA	6.“Accepting life”/ PPs
7.“Keeping my life active”/ BA	7.“Achieving a full life”/ PPs
8.“From now on, what else…?”/Relapse prevention	8.“From now on, what else…?”/Relapse prevention

Note. Modules 1, 2, 3, and 8 are included in all three conditions

It is important to mention that the three protocols have the same number of modules (eight) and a similar number of words: IGc: 35.123; IBAc 35.492 and IPPc: 39.002 (M: 36541; SD: 2139.003).

The three treatment protocols include a “Welcome” module that explains information about the treatment protocol and its purposes. Furthermore, it explains the main recommendations for obtaining the most benefit from it. After this “Welcome” module, the patients access the pre-treatment assessment online questionnaires. After the pre-treatment assessment, participants start the intervention modules. Table 2 shows the structure of each module. The modules include exercises to practice each technique and skill. Furthermore, at the end of each module, there is a post-module assessment to evaluate depression, anxiety, and positive/negative affect. When participants complete the eight treatment modules, they perform the post-treatment assessment, which is also integrated in the web system (the same self-report questionnaires as in the pre-treatment assessment, plus the treatment satisfaction scale).

**Table 2:** Structure of each module

Structure of each module	
1. Questions related to the previous module	
2. Specific contents of the module	
3. Exercises related to the content of the module	
4. Homework tasks telling them to work on what was presented in the module	
5. Post module assessment: depression, anxiety, positive and negative affect	

An important function of the program is that the therapists have access to all the information participants provide during the treatment, so that if the patient’s condition gets worse they can receive an alert. These alerts are generated by the system when a high risk of suicide is detected. Then, an email is sent to the clinical team so that the therapist can contact the patients and make better decisions to protect and help them.

Regarding the use of the intervention, participants will progress sequentially through the program at their own rhythm, but they will be informed that they will receive the most benefit from the intervention if they do about one module every 2 weeks. This is the time stipulated to complete each module and practice the techniques and strategies learned. The participants will be informed that they have a maximum of 16 weeks to complete all eight modules. As the intervention progresses, when they finish one module, they will be allowed to look it over again if they so desire.

All the modules will be on a web platform developed by our research group (https://www.psicologiaytecnologia.com/). It was designed for optimal use on a tablet or a PC and to optimize the understanding of the modules’ content using different multimedia elements such as audio, vignettes, video, etc. The web platform has different transversal tools that accompany the person throughout the entire intervention process (Table 3).

**Table 3:** Transversal tools of the web platform

“Home”	This tool is the starting point of the protocol, appears on the main menu, and is used to access the other sections of the protocol. It also shows the progress through the treatment.
“Calendar”	In this section, the participant can know where he/she is in the program. This tool also shows the days on which the participant has accessed the program, as well as both pending and achieved tasks.
“Review”	This section is used by participants to review the treatment modules already completed. This tool allows them to have access to the different modules as often as they wish.
“How am I”	This section offers several graphs that make it possible to monitor the participant’s progress. It provides feedback to participants about their activity level, emotional distress, and positive and negative emotionality.
“Diary register”	The objective of the “Diary register” is to collect data every day about different variables (activity level, emotional distress, positive and negative emotionality) and show them graphically on the “How am I” tool.

### Support

In the intervention conditions, the participants will receive human and ICT support.

Regarding human support, one trained predoctoral student in our group will make several brief phone calls at four points in time:

- *An initial telephone session:* to explain the characteristics of the RCT to the participant and administer the clinical diagnostic interview and find out whether s/he fulfills the inclusion criteria.
- *An initial phone call in the “Welcome” module*: encouraging patients to start the program, do one module every 2 weeks, and do the homework tasks in each module. This phone call will take place when the participants do the pre-treatment assessments.
- *One brief phone call (maximum of 10 min) when the participants reach the mid-point of the intervention (module 4)*: 1) to ask the participants about any doubts or difficulties regarding the use of the program and help them; 2) to remind them that they can review the content of each module; 3) to emphasize the importance of doing the tasks in each module and practice the strategies they learn; 4) to motivate patients to continue with the program and positively reinforce them for engaging in the intervention; and 5) to remember that the best way to experience the intervention is by doing one module every 2 weeks.
- *A final phone call after the post-assessment:* to ask them their qualitative opinion about the intervention and remind participants that they will be allowed to use the intervention program any time they want to during the study period, and that we will contact them to do the follow-up assessment.

ICT support will consist of multiple-choice questions about the module’s contents in order to provide the participant with the correct feedback for their responses and a detailed explanation. Furthermore, the participants will receive an automated email encouraging them to continue with the modules if they have not accessed the program for a week. In addition to this automated support, the program offers continued feedback to users through the transversal tools described earlier.

### Instruments

#### Diagnostic interview

Mini International Neuropsychiatric Interview Version 5.0.0 (MINI) [61]. It is a structured diagnostic interview for DSM-IV and ICD-10 psychiatric disorders. It was designed to be used by clinicians or even by nonclinical personnel after brief training, and it has an administration time of approximately 15 min. The MINI has excellent interrater reliability (K = .88–1.00), and it has been translated and validated in Spanish [62].

#### Primary outcomes

##### Depression

Beck Depression Inventory (BDI-II) the BDI-II is a 21-item self-report multiple-choice inventory that is widely used to detect and assess the severity of depressive symptoms. The items, scored on a scale from 0 to 3, cover the different symptoms characterizing major depression disorder in the DSM-IV [57], such as sadness, pessimism, past failure, loss of pleasure, guilty feelings, punishment feelings, suicidal thoughts or wishes, etc. The scores on the scale range from 0 to 63. The internal consistency of the BDI-II is high (alpha = 0.76 to 0.95), and for the Spanish version of the instrument (alpha = 0.87), for both general and clinical populations (alpha = 0.89) [63].

##### Positive and negative emotionality

Positive and Negative Affect Scale (PANAS) the PANAS [64] consists of two 10-item mood scales that assess two independent dominant dimensions of affective structure: positive affect (PA) and negative affect (NA). Each scale ranges from 10 to 50. The PANAS is brief, reliable, and has shown excellent internal consistency (alpha between 0.84 and 0.90) and convergent and divergent validity. The Spanish version has also shown high internal consistency (α = 0.87 and 0.89 for PA and NA in men, respectively, and α = 0.89 and 0.91 for PA and NA in women, respectively [64].

##### Secondary outcomes and post-module measures

Connor-Davidson Resilience Scale (CD-RISC) the CD-RISC [65] is a brief scale that consists of 25 items. The person must indicate to what extent each statement has been true for him/her in the past month on a scale from 0 to 4, where 0 =“has not been true at all” and 4=“true almost always”. The total scores range from 0 to 100; higher scores indicate greater resilience. Previous studies have shown that the CD-RISC has good internal consistency (Cronbach alpha above 0.70) [65].

##### Overall Depression Severity and Impairment Scale (ODSIS)

The ODSIS [66] is a brief, 5-item self-report measure for assessing the frequency and severity of depression, as well as functional impairments in pleasurable activities, work or school interference, and social relationship interference associated with depression. The items are scored on a scale from 0 to 4, and they function similarly across clinical and nonclinical samples. The ODSIS has shown excellent internal consistency (Cronbach’s alpha between .91 and .94) and good convergent/discriminant validity [66].

##### Perceived Stress Scale (PSS)

The PSS [67] is a self-report instrument that assesses the level of stress perceived in the past month. It consists of 14 items scored on a scale ranging from 0 (never) to 4 (very often). The total score range varies between 0 (minimum perceived stress) and 56 (maximum perceived stress). In the present study, a 4-item PSS (PSS-4) was used. This PSS-4 was introduced as a brief version for situations requiring a very short scale or telephone interviews [68]. It has been validated in different studies, showing an internal consistency reliability of 0.76–0.82 [69].

##### Overall Anxiety Severity and Impairment Scale (OASIS)

The OASIS [70] is a 5-item self-report measure, rated from 0 to 4, that can be used to assess the frequency and severity of anxiety disorders, multiple anxiety disorders, and subthreshold anxiety symptoms. The scale also provides measures of functional impairments in pleasurable activities, work or school interference, and social relationship interference associated with anxiety symptoms. The OASIS has demonstrated strong psychometric properties with good internal consistency (alpha = 0.80), test-retest reliability (K = 0.82), and convergent validity [70–72].

##### Quality of life Inventory (QLI)

The QLI [73] is a brief self-report questionnaire used to measure the perceived quality of life in different areas. The inventory consists of 10 items scored on a scale ranging from 1 to 10. It is calculated by averaging the 10 items, and the maximum score is 10. The QLI assesses aspects related to physical well-being, psychological well-being, self-care and independent functioning, occupational functioning, interpersonal functioning, social emotional support, community and services support, personal fulfillment, spiritual fulfillment, and overall quality of life. The QLI has shown excellent internal consistency (between 0.90 and 0.92), test-retest reliability (0.87), and discriminant validity. The Spanish validation of the QLI [74] has also demonstrated good test-retest reliability (0.89) and discriminant validity.

##### Pemberton Happiness Index (PHI)

The PHI [75] is a brief instrument to measure different domains of well-being (i.e., general, hedonic, eudemonic, and social). It contains 11 items rated on a scale where 0 = strongly disagree and 10 = strongly agree. The reliability of this scale is quite satisfactory (alpha = 0.89) [75].

##### Enjoyment Orientation Scale (EOS)

The EOS [76] is a 6-item self-report measure that assesses the extent to which participants try to be receptive and make an effort to be engaged in pleasant things (anticipatory pleasure). This scale is very related to the behavioral activation system that is believed to regulate appetitive motives. The items on the EOS are rated on a Likert scale from 1 (“strongly disagree”) to 7 (“strongly agree”) [76].

##### Environmental Reward Observation Scale (EROS)

The EROS [77] is a brief, reliable, and valid measure of environmental reward. It consists of a 10-item Likert measure rated from 1 (“strongly disagree”) to 4 (“strongly agree”), with the total score representing the sum of the 10 items. The Spanish EROS is internally consistent (alpha = 0.86) and valid [77].

##### Acceptance, satisfaction and usability outcomes

Expectation of treatment scale and opinion of treatment scale these two scales are adapted from Borkovec and Nau [78]. Each scale contains 5 items, regarding whether the treatment is logical, treatment satisfaction, the treatment’s utility for other psychological problems, and the treatment’s usefulness for the patient’s specific problem. The expectation scale is administered at the post-module 2 assessment, when the treatment has been explained to the participants, and the opinion scale is administered at the end of the treatment, with the aim of assessing satisfaction. Our group has used this questionnaire in several research studies [51, 79].

**System Usability Scale (SUS)**

The SUS [80] is a brief, reliable scale for measuring the usability of a program. It consists of a 10-item questionnaire with 5 response options, from 0 (“strongly disagree”) to 4 (“strongly agree”). Its purpose is to collect the user’s opinion about the usability of the system, and unacceptable usability may indicate that the user has had technical difficulties with the program. The SUS adjective rating scale (from “Worst imaginable” to “Best imaginable”) will be used to provide a qualitative comparison of usability scores [81].

The study measures and area and time of assessment are summarized in Table 4.

**Table 4:** Study measures, assessment area and time of assessment

Outcomes	Concept	Instrument	Time of assessment			
			BL	Post-T	Post-M	FUPs (3, 6, 12)
Diagnostic interview	Diagnosis	MINI Neuropsychiatric Interview	X	X		X
Primary	Depression	BDI-II	X	X		X
	Positive and Negative affect	PANAS	X	X	X	X
Secondary	Resilience	CD-RISC	X	X		X
	Depression	ODSIS	X	X	X	X
	Perceived Stress	PSS	X	X		X
	Anxiety	OASIS	X	X	X	X
	Quality of life	QLI	X	X		X
	- Well-being	PHI	X	X		X
	- Anticipatory pleasure	EOS	X	X		X
	- Environmental reward	EROS	X	X		X
	Acceptance, satisfaction and usability outcomes					
	- Expectation of treatment	Expectation of Treatment Scale	X			
	- Opinion of treatment	Opinion of Treatment Scale		X		
	- Usability	SUS		X		

Note: *BL* Baseline, *Post-T* Post-treatment, *Post-M Post Module*, *FUPs (3,6,12)* 3, 6, and 12-month follow-ups, *BDI-II* Beck Depression Inventory-II, *CD-RISC* Connor-Davidson Resilience Scale, *PANAS* Positive and Negative Affect Scale, *ODSIS* Overall Depression Severity and Impairment Scale, *PSS* Perceived Stress Scale, *OASIS* Overall Anxiety Severity and Impairment Scale, *QLI* Quality of Life Index, *PHI* Pemberton Happiness Index, *EOS* Enjoyment Orientation Scale, *EROS* Environmental Reward Observation Scale, *SUS* System Usability Scale

**Sample size and power calculations**

The a priori sample size determination was performed based on the main objective of this investigation. Following Cohen’s (1988) guidelines, we assumed a proportion of explained variance of low magnitude for the interaction between the type of intervention and the measurement occasion, that is, 0.01. For a significance level of 5%, statistical power of 80%, assumed sphericity, and correlation of .7 between repeated measures (following Rosenthal’s 1991 recommendation), the total sample size needed was 147 participants (49 per intervention group). To this figure, an additional 30% was added to anticipate potential dropouts. Therefore, the total sample size of the study was 192 participants (64 per intervention group). Sample size calculations were carried out with the statistical program G*Power 3.1.9.2 [82].

### Statistical analysis

Per protocol and Intention-to-treat (ITT) analyses will be performed, following the CONSORT and CONSORT-eHealth recommendations [56, 83]. Between-group differences in baseline clinical and socio-demographic characteristics will be explored using analysis of variance (ANOVA) for continuous data, and chi-square tests (χ2) for categorical variables. Both normality and multinormality assumptions will be assessed by applying the Kolmogorov-Smirnov (K-S) test, skewness and kurtosis indexes, and histogram and Q-Q plots. Levene’s test will be conducted to check homoscedasticity assumptions for equality of variances, and Mauchly’s test to explore sphericity assumptions. Missing data patterns will be assessed, and whether missing data are missing completely at random (MCAR) will be checked with Little’s MCAR test [84]. The ITT principle will be applied for primary and secondary outcomes collected at post-treatment, and at the 3, 6, and 12-month follow-ups. In this regard, the maximum likelihood (ML) method using the Expectation Maximization (EM) algorithm will be used to deal with missing data, due to its flexibility in repeated-measures ANOVAs in handling missing data appropriately (i.e., [85, 86]. Nevertheless, several approaches will also be considered and assessed using sensitivity analyses in order to apply the most robust and adequate method based on both missing data patterns and literature recommendations (i.e., [87, 88]. Repeated analysis of variance (rm-ANOVA) will be performed to explore the main and interaction effects of the treatments on all the primary and secondary outcomes. Significant effects will be followed up by pairwise comparisons, such as the Tukey procedure when the homoscedasticity assumption is met, and the Games-Howell procedure if this assumption is not met. Effect sizes (Cohen’s *d*) and their respective 95% confidence intervals (95% IC) will be calculated and reported for within- and between-group comparisons, according to authors’ recommendations [89–91]. In addition to ITT analyses, per protocol analyses will also be conducted. It is true that these analyses suffer from selection bias, but they will allow us to reach conclusions about the efficacy of the treatment in patients who complete all the intervention modules [92].

For acceptability and usability measures, data analysis will be based on completers. Separate multivariate analysis of variance (MANOVA) for expectations, satisfaction, and usability will be performed, where all items are entered into the MANOVA as dependent variables, and the experimental group as a fixed factor (independent variable).

Finally, multiple regression analysis and mediation analysis will be conducted to explore potential predictors and mediators of depressive symptoms. Mediation analyses will be performed through bootstrap regression analysis using the Preacher and Hayes (2004) approach. All statistical analyses will be conducted using IBM SPSS Statistics for Windows, version 23.

We will review the state of the art analytical methodology for RCT before performing the analyses of the data, in order to use the best statistical analysis procedure. Thus, there may be some variations in the statistical analysis procedures.

## Discussion

This paper describes the protocol for a randomized controlled dismantling study of an Internet-based intervention for patients with mild to moderate depressive symptoms. One of the main aims of this study is to evaluate the efficacy of a complete Internet-based intervention for depressive symptoms (including motivation, cognitive restructuring, BA, PPs, and Relapse prevention), the same Internet-based intervention without the PPs component, and the same Internet-based intervention without the BA component, all of them administered over the Internet to patients with mild to moderate depressive symptoms. These three intervention protocols with different therapeutic components will be tested in order to explore the specific contribution of each therapeutic component involved in the treatment of depression and better understand how and why therapies lead to change [20].

Although a large number of psychological interventions have been shown to be effective in the treatment of depression, the specific influence of each therapeutic component is still unknown [15]. Thus, it is crucial to delimit the influence that the specific therapeutic components of psychological interventions can have.

Furthermore, current psychological treatments for depression focus largely on reducing excesses in negative affect rather than on specifically improving deficits in positive affect and well-being [36, 93]. It is well known that depression often involves low levels of positive affect [22, 93] that increase the severity of the problem [94]. Furthermore, people with high levels of positive affect tend to have better well-being and psychological and physical health [95].

In this regard, it is important to include positive affect as an essential target of the treatment by considering well-being and positive functioning to be core elements of the intervention.

Recent literature shows that PPs techniques might have an impact on the decline in clinical symptomatology [22, 36, 96, 97]. These PPs (Well-being Therapy; Quality of Life therapy; Positive Psychotherapy, and Strengths based Counseling) are based on the hypothesis that it is possible to treat depression not only by focusing on decreasing the negative symptoms, but also by directly and primarily building positive emotions and promoting psychological strengths and a meaningful life [27, 31]. Recent findings suggest that explicitly focusing on positive emotions efficiently improves depressive symptoms and helps to achieve more profound change in positive functioning measures [98–100]. However, the specific contribution of these PPs in the treatment of depression has scarcely been studied. In fact, in a recent comprehensive systematic review and meta-analysis of dismantling studies of psychotherapies for adult depression, no study is included that dismantles an intervention with a PPs-based component, and none with an Internet-based intervention [15].

The dismantling design of the current study will allow us to explore the contribution of each main treatment component. More specifically, it will allow us to know how PPs and emotional regulation strategies centered on positive affect work, resulting in a significant shift to optimize treatments for depression. In addition, with the current study, it will be possible to analyze the mediators of changes in depressive symptoms and the acceptability of each intervention.

Moreover, this study is consistent with one of the most important challenges within the field of the treatment of depression, which is the design of new ways to apply treatments to maximize their therapeutic efficiency. Undoubtedly, the use of technology and the Internet can help to achieve this goal and contribute to the dissemination and accessibility of evidence-based treatments. Furthermore, another advantage of the Internet-based interventions is the possibility of making online assessments. Research results encourage the use of online questionnaires because they offer advantages over traditional data collection strategies [101, 102]. Some of these advantages are that missing data can be handled better, and the assessment is easy and immediate [101] and can take place right after the patients finish a psychological component. This could make it easier to know the specific contribution of each component throughout the intervention process.

In sum, this study has several strengths. To the best of our knowledge, this is the first randomized dismantling intervention study for depression with the aim of exploring the contribution of a PPs component and the BA component in an Internet-based intervention. The three protocols are online interventions, helping to reach many people who need psychological treatments and would not otherwise have access to them. Furthermore, acceptability and usability measures will be included in order to assess the intervention’s feasibility and acceptance, in addition to an efficacy study. Finally, potential mediator mechanisms will be explored to identify the variables that lead to changes in depressive symptoms.

We are aware that this study has limitations. One of them is that the number of BA and PPs modules in the *IBAc* and *IPPc* is higher than in the *IGc*, although the clinical content is the same in the global protocol and in the protocols for the BA and PPs components. The reason for this is that the three protocols contain the same total number of modules (8 modules per each) and a similar number of words. We give all the patients the same amount of time to do the intervention to try to control this. Another limitation is that the dropout rates are expected to be high (around 30%) [103]. Efforts will be made to minimize the dropout rates by providing ICT and human support. Approaches to handling missing data will be considered and assessed using sensitivity analyses in order to apply the most robust and adequate method, based on both the missing data patterns and recommendations found in the literature (i.e., [87, 88]. In addition, difficulties in the recruitment phase will be considered.

In summary, this study extends the current literature about Internet-based interventions for depression. If positive results are achieved, they may have an important impact by providing better knowledge about the core elements of evidence-based treatments for depression.

## Abbreviations

AES: Advanced Encryption Standard
ANOVA: Analysis of Variance
BA: Behavioral Activation
BDI-II: Beck Depression Inventory-II
BL: Baseline
CBT: Cognitive Behavior Therapy
CONSORT: Consolidated Standards of Reporting Trials
EM: Expectation Maximization
EOS: Enjoyment Orientation Scale
EROS: Environmental Reward Observation Scale
FU: Follow-Ups
IBAc: Internet-based Behavioral Activation protocol condition
IGc: Internet-based Global Protocol condition
IPPc: Internet-based Positive Psychotherapy protocol condition
ITT: Intention-to-treat
MANOVA: Multivariate Analysis of Variance
MCAR: Missing Completely at Random
MINI 5.0.0: Mini International Neuropsychiatric Interview Version (5.0.0)
ML: Maximum Likelihood
OASIS: Overall Anxiety Severity and Impairment Scale
ODSIS: Overall Depression Severity and Impairment Scale
PANAS: Positive and Negative Affect Scale
PHI: Pemberton Happiness Index
Post-T: Post-Treatment
PPs: Positive Psychotherapeutic strategies
PSS: Perceived Stress Scale
QLI: Quality of Life Index
RS-14: Resilience Scale-14
SPIRIT: Standard Protocol Items: Recommendations for Interventional Trials
SUS: System Usability Scale
